# Nocturnal spawning as a way to avoid egg exposure to diurnal predators

**DOI:** 10.1038/s41598-018-33615-4

**Published:** 2018-10-18

**Authors:** Marek Šmejkal, Allan T. Souza, Petr Blabolil, Daniel Bartoň, Zuzana Sajdlová, Lukáš Vejřík, Jan Kubečka

**Affiliations:** 1 Biology Centre of the Czech Academy of Sciences, Institute of Hydrobiology, České Budějovice, Czech Republic; 2 Biology Centre of the Czech Academy of Sciences, SOWA, České Budějovice, Czech Republic

## Abstract

Animals that do not provide parental care have to secure the survival of their offspring by ensuring a safe reproductive environment or smart timing tactics. Nocturnal spawning behaviour of many fish species is an example of the latter behaviour in the animal kingdom and is hypothesized to provide a survival advantage to the eggs spawned during the night. In order to test the efficiency of the smart timing tactics in a freshwater fish, a study was carried out of the interaction of the rheophilic spawner (asp *Leuciscus aspius*) and the predator of its drifting eggs (bleak *Alburnus alburnus*) using passive telemetry. According to a model based on acquired data, asp laid 63% of its eggs at night, while vision-oriented bleak was present in 92% of the time during the day. This study gives support to the predator avoidance hypothesis, which expects animals to reproduce in a period when the probability of offspring predation is at its lowest.

## Introduction

The threat of predation is a driving force in many behavioural decisions in the animal kingdom. Animals optimize their feeding and mating behaviour based on the level of risk of being attacked by their natural enemies^1–3^. In many cases, it is not the individuals themselves, but their offspring, who may suffer the consequences of the parent’s anti-predation decisions^4–6^. Hence, offspring potential mortality should be evaluated by their parents in order to maximize parent’s lifetime fitness.

While the threat of predation may be minimized by parental care, many species do not protect their offspring and have to use alternative strategies to avoid high offspring mortality^7,8^. In the case of fish, only one quarter of fish families have evolved some sort of parental care, while no species from remaining families protect their eggs. In the latter case, fish are hypothesized to use three different strategies to reduce egg mortality^9^. The predator swamping hypothesis postulates that fish synchronize spawning into a short period of time and use limited feeding capacity of egg-eating predators^4,10,11^. The second hypothesis suggests that fish time their reproduction periods to when egg-eating predators are already satiated by their regular non-egg diet^9,12^. The third hypothesis explaining spawning behaviour by predator avoidance postulates that fish focus their reproduction during periods when egg-eating predators are inactive and/or cannot detect the eggs. This strategy may be used in instances where the eggs are already dispersed or hidden in the environment when the egg-eating predators resume their feeding regime^12–14^. Often, it is hard to discriminate among the three hypotheses since they are not mutually exclusive: dusk and night spawning makes searching for eggs harder, but predators may be already satiated from their regular diet, hence their motivation for twilight and nocturnal feeding may be low.

To avoid high predation risk from diurnal predators, animals often shift their feeding and reproductive activity to crepuscular and nocturnal periods, when the mortality risk is considerably lower^2,15^. For instance, many coral reef fish species spawn at these periods despite their more normal diurnal feeding activity^12–14,16^. However, many of these observations of spawning are limited by visual methodologies and fish behaviour was studied during daytime and crepuscular periods only.

The aim of this study was to discriminate between the above-mentioned three hypotheses that might be exhibited by fish to reduce mortality of the eggs spawned. This phenomenon was studied in two sympatric freshwater fish species, namely, asp *Leuciscus aspius* and bleak *Alburnus alburnus*^17^. Asp is a large predator cyprinid species inhabiting Central and Eastern Europe that spawns in early spring in fast flowing lotic waters^18–20^. The spawning fish release eggs in the water column and they are carried by the water current for a few meters before attaching onto the substrate^21^. Bleak forage on prey such as zooplankton, water insects and drifting fish eggs in the asp spawning season, and do not utilize benthic food sources including eggs attached to the substrate^18,22–24^. Both species are visually-oriented daytime active species during the summer season with little or no nocturnal activity^24–26^.

To find support for the above hypothesis on behavioural adaptations to avoid high egg mortality, passive telemetry systems were used which monitored the spawning ground during the asp reproductive season. Asp eggs and bleak presence were modelled based on telemetry data and compared to see whether 24-hour distribution of asp eggs spawned corresponds with the presence of bleak on the spawning ground. It was expected that (1) egg distribution peaking in the same time as bleak presence supports the predator swamping hypothesis, (2) tendency to nocturnal egg peak and absence of bleak nocturnal activity supports the predator avoidance hypothesis but does not fully exclude the predator satiation hypothesis. Based on goodness of fit between asp eggs and bleak presence, the authors inferred which hypothesis, if any, was supported by the collected data.

## Materials and Methods

### Study site

The study site is located in the main tributary of the Želivka Reservoir, 49°578497′ N, 15°251671′ E, Czech Republic. More than 2000 asps migrate yearly from the reservoir (39 km long, 1602 ha) to reproduce in the early spring (end of March to mid-April) at a 100 m long and 20 m wide spawning ground^19,27^. Bleak enters the asp spawning ground and forage on drifting asp eggs, causing considerable mortality of asp drifting eggs during the day, before the eggs attach onto the stones and pebbles present in the spawning ground^22^.

### Fish capture and tagging procedure

Fish were captured using an electrofishing boat (electrofisher EL 65 II GL DC, Hans Grassel, Schönau am Königsee, Germany, 13 kW, 300/600 V) during spawning seasons 2014 and 2015 (asp only), 2016 (asp and bleak) and 2017 (bleak only). Subsequently, the fish were anaesthetized with MS-222, and their total length (TL), weight and sex (in asp only) were recorded. In asp, recognition of males was based on milt release, breeding tubercles and a slender body, whereas females had no tubercles and a robust body. Asp scales were taken for age determination and future growth modelling (further details provided in the section Modelling distribution of asp eggs). After fish anesthetization, a 4–5 mm vertical incision was made 3 cm posterior to the pelvic fin, and a 32 mm PIT tag for asp (Oregon RFID, half-duplex, diameter 3.65 mm, weight 0.8 g, ISO 11784/11785 compatible) or a 23 mm PIT tag for bleak (Oregon RFID, half-duplex, diameter 3.65 mm, weight 0.6 g, ISO 11784/11785 compatible) was inserted into the body cavity. No sutures were used to close the incision according to cyprinid tagging methodology^28,29^. The tagged individuals were released immediately after recovery from anaesthesia. Overall, 373 males and 261 females asp tagged between 2014 and 2016 arrived at the spawning ground, with sizes varying between males (SL ± standard deviation SD, 470 ± 53 mm) and females (495 ± 80 mm). In the case of bleak, 35 and 23 unique bleak individuals (120 ± 7 mm and 119 ± 10 mm) arrived at the spawning ground in 2016 and 2017, respectively.

### Fish passive telemetry

**Figure 1:**
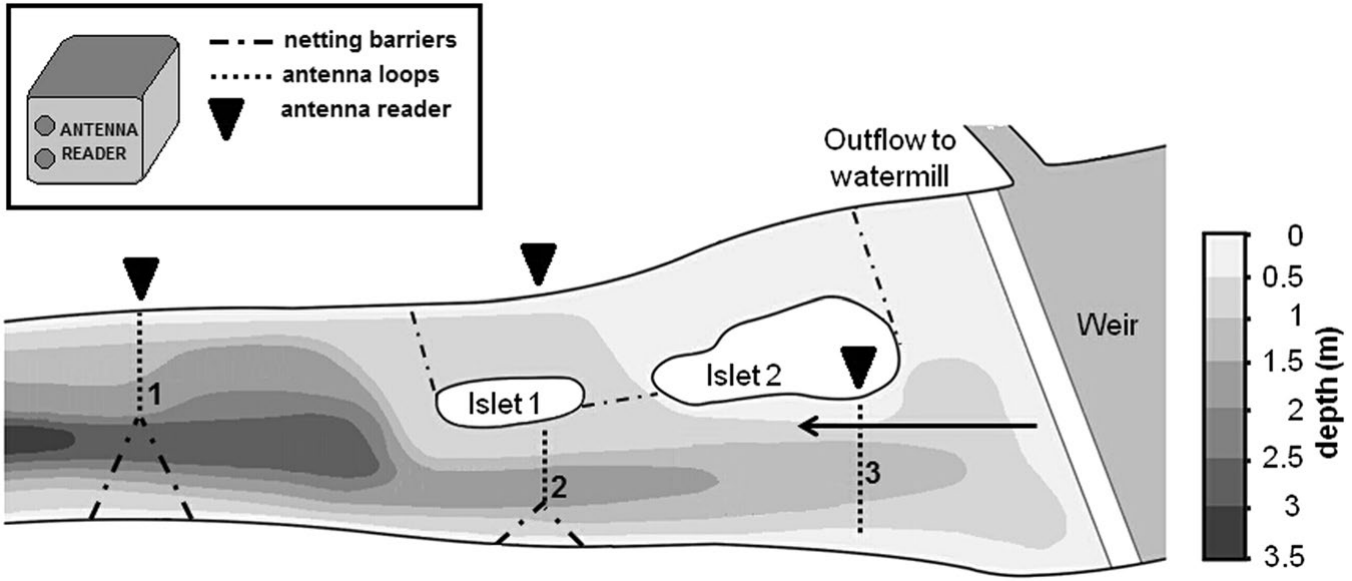
Schematic representation of the monitored asp spawning ground with the placement of three antenna systems indicated by numbers. Arrow shows the direction of flow.

The presence of the tagged individuals was recorded via passive telemetry systems (Oregon RFID, LF HDX RFID readers) and altogether, 276 and 579 individuals were detected in 2016 and 2017, respectively. When a tagged fish passes the antenna, the PIT tag is energized and emits an individual 12-digit code that is recorded and stored together with the date and time. The reader recording frequency was set to 10 energize/receive cycles s^−1^, meaning that fish are recorded every tenth of a second if present in the antenna scanning range. Three synchronized systems were installed in the main tributary of the Želivka Reservoir 50 m apart. The river topography allowed cover of only half of the river, so fish were guided to swim through the 10-m wide antennas using barriers (Fig. 1). Along with fish monitoring, a data logger (TidbiT v2, Onset, USA) measuring water temperature once every hour was placed at the tributary.

### Ethics

The field sampling and experimental protocols used in this study were performed in accordance with the guidelines of and with permission from the Experimental Animal Welfare Commission under the Ministry of Agriculture of the Czech Republic (Ref. No. CZ 01679). All methods were approved by the Experimental Animal Welfare Commission under the Ministry of Agriculture of the Czech Republic.

### Telemetry data processing

The data obtained for the two species had to be treated differently due to differences in their lifespan. In the case of long-lived asp, fish tagged and detected in the same spawning season were excluded from the analysis and their reproductive behaviour was analysed a year after tagging^19^. In the short-lived bleak, data obtained one day after tagging or later were analysed (bleak start to be present at the spawning ground approximately at the beginning of April, which limits the days of effective tracking to about two weeks).

Detections by any antenna were used as a proxy for individual presence at the asp spawning ground (Fig. 1)^19^. Fish were considered present if individual detections were logged within a two-hour period, otherwise the last detection was assumed to be fish departure. Because the primal purpose of the study was to compare presence of eggs and bleak, tagged male asp were excluded computations were made only with female asp and bleak. Due to a reproductive system where males are always in excess, females are never limited by the number of males^19^.

### Modelling the distribution of asp eggs

To model the presence of the drifting stage of asp eggs, it was assumed that the female presence on the spawning ground is associated with spawning activity due to their very short (on the average six hour) visits^19^ and observations that recorded higher fish abundance and spawning frequency at night (Supplementary material 1). Female asp was tagged at least one year prior the records of the spawning activity in order to observe their natural behaviour without any influence of tagging and electrofishing^30,31^. The amount of eggs based on its recorded length during tagging could not be directly estimated due to the growth increment in following year. Hence, female asp length was modelled based on age determined from scale readings of their first capture and the individual-based von-Bertalanffy growth equation (Supplementary material 2). In fish that have been recaptured in the year of the behavioural study, their predicted length was corrected based on their true length increment.

Once the length of fish was estimated for the duration of the study, female weight (*W*) was computed from the equation of asp female length-weight relationship (1):

$${\rm{W}}={\rm{a}}\times {{\rm{TL}}}^{{\rm{b}}}[{\rm{g}}],$$

with coefficient a = 0.00744 and b = 3.046. Coefficients were obtained from the asp database using non-ovulating females for the computation.

The fecundity of individual fish for a given size was estimated based on gonadosomatic index of asp^32,33^. For the modelling purposes of the drifting egg availability, the number of eggs produced was distributed evenly over the time female spent on the spawning ground.

### Statistical analysis

A Linear mixed model using Sattertwaite approximations to degrees of freedom was used to determine what variables (fixed effects: period of the day (day vs. night), day of the year, bleak presence and temperature; random effects: year) influence the number of asp eggs. To cope with inter-annual variations on the number of tagged fish, the year was used as a random effect, given that it is not expected that the fish behaviour would change between years.

In order to determine whether the above-mentioned variables drive bleak presence in the same direction, a linear mixed model using Sattertwaite approximations to degrees of freedom explaining bleak presence was used with variables (fixed effects: period of the day, day of a year and number of eggs; random effects: bleak identity). Stepwise selection on best models was performed using Akaike Information Criterion. Models were run using the lme4 library^34^ implemented in the R software^35^.

Prior to the statistical analysis, the data was centred and scaled to avoid bias due to dissimilar range of variation among variables. Correlation between paired samples was tested using Kendall’s *tau* rank correlation. Homoscedasticity was checked by visual inspection of the model residuals^36^.

## Results

Altogether, 98 and 230 asp females arrived and were used for the egg presence modelling in 2016 and 2017, respectively. Distribution of bleak presence was based on 35 and 23 individuals that were present on the spawning ground in 2016 and 2017, respectively.

**Figure 2:**
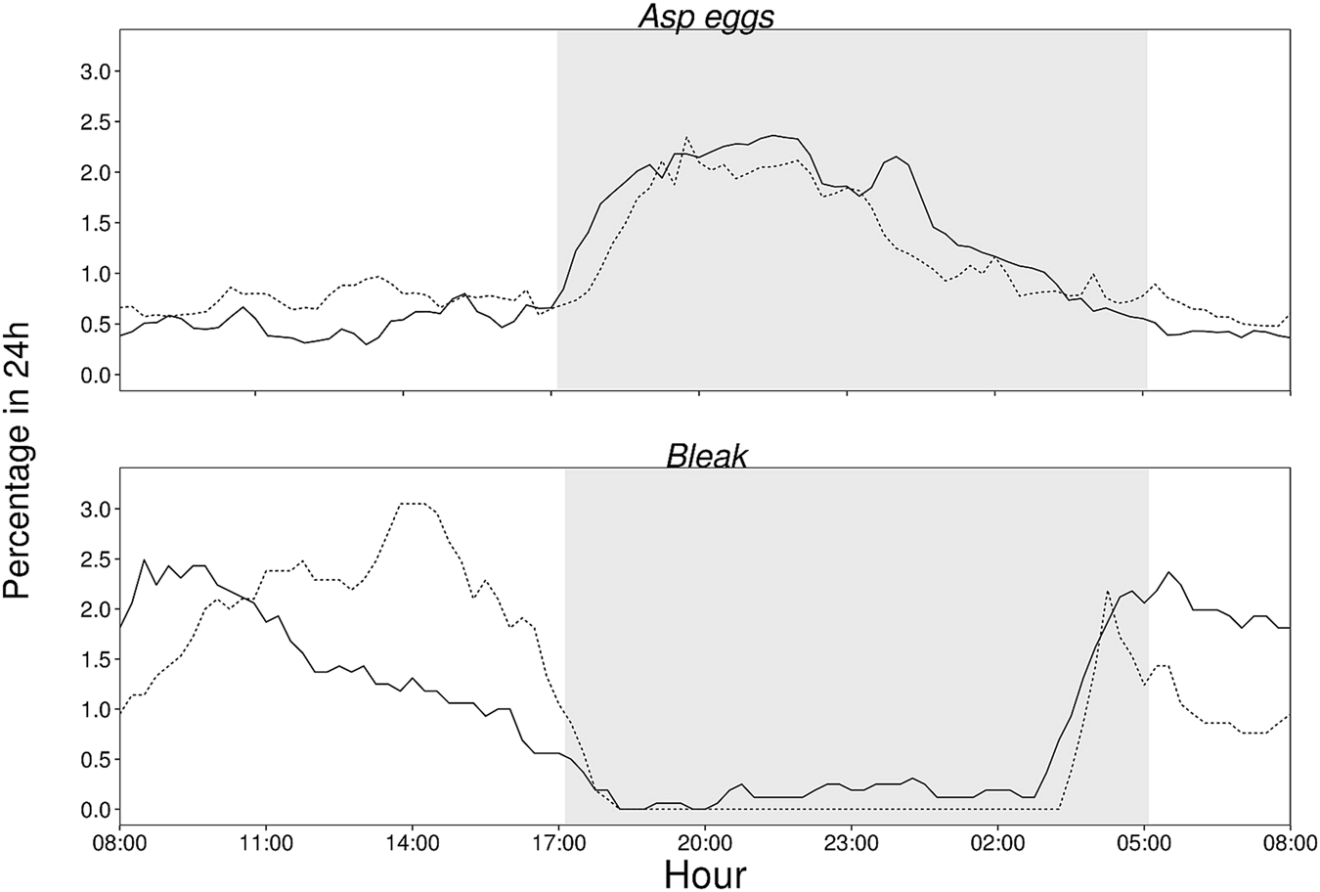
Distribution of the asp eggs and bleak presence in a 24-hour period (UTC time zone) summarized from both 2016 (dashed line) and 2017 (dotted line). Figure is based on 98 and 230 asp females and 35 and 23 bleak individuals that arrived at the spawning ground in 2016 and 2017, respectively. Asp eggs were more abundant at night (63%), while bleak was from 92% present during the day, suggesting a mismatch between egg presence and their predator.

The number of eggs was significantly dependent on the period of the day (63.07% ± 23.06 at night), day of the year, temperature (more eggs present in relatively higher temperatures) and their presence in any given day was correlated with bleak presence (Table 1, Fig. 2). The latter relationship between asp egg and bleak presence could be related to bleak egg-foraging behaviour.

**Table 1:** Outputs from the linear mixed effects model on the drifting egg presence in the spawning ground and its predictors.

	Estimate	Std. Error	df	t-value	p-value
intercept	−0.200	0.306	2	−0.662	0.576
date	−0.541	0.015	4439	−47.047	<0.001
bleak presence	0.026	0.009	4439	2.645	<0.01
period of the day	0.400	0.021	4439	18.875	<0.001
water temperature	0.183	0.014	4439	12.712	<0.001

Random effects on intercept were related to the egg predator - bleak identity. Marginal test: r^2^ = 0.33; conditional test: r^2^ = 0.53.

Bleak was present at the spawning ground predominantly during the day and left for the night (percentage of detected individuals at night 8.45 ± 19.95%). According to the linear mixed model, significant variables predicting bleak presence were period of the day – bleak preferred the day to the night period, season - bleak arrived more towards the end of the spawning season and when eggs were more abundant at the spawning ground (Table 2, Fig. 2). Temperature was not significant in the model and was excluded.

**Table 2:** Outputs from the linear mixed effects model on the bleak presence in the spawning ground and its predictors.

	Estimate	Std. Error	df	t-value	p-value
intercept	0.118	0.044	75.75	2.705	<0.01
date	0.133	0.030	78.32	4.505	<0.001
eggs	0.046	0.019	175.39	2.436	<0.05
period of the day	−0.236	0.049	83.15	−4.806	<0.001

Random effects on intercept and slope were related to bleak identity. Marginal test: r^2^ = 0.03; conditional test: r^2^ = 0.11.

## Discussion

The data demonstrated that almost two thirds of eggs were laid at night, while passive telemetry revealed that bleak entered the asp’s spawning ground almost exclusively during the day. Due to such behaviour, bleak missed the opportunity to forage at a time when the majority of eggs were deposited. The lack of overlap between egg and bleak presence in both studied seasons suggests that there exists an apparent asynchrony between egg and bleak presence.

Findings in this study support the predator avoidance hypothesis, since asp predominantly laid their eggs at night, when bleak do not forage. Bleak telemetry data demonstrated that most bleak left the spawning ground before sunset and arrived again shortly before sunrise, confirming the absence of strong predator pressure at night. Both investigated species are active during the day and at twilight in the summer season^24,26^ and bleak demonstrated this pattern consistently in the studied period.

The spawning ground is characterized by a fast water current, and therefore it requires considerable effort to maintain position in the spawning ground^19^. The reason for leaving the spawning ground is thought to be due to the high effort required in order to remain in the spawning ground combined with low or no foraging at night. Therefore, bleak migrated downstream to standing water when foraging on the asp eggs became inefficient. Alternatively, the denser presence of large asp on the spawning ground at night may have caused avoidance by bleak. However, the latter explanation may not hold true, since asp do not forage on bleak until the end of spawning period^22^ and therefore do not represent a threat for egg eating bleak.

The predator satiation hypothesis would expect that bleak became satiated during the day and therefore departed from the spawning ground in the evening. However, gut content analyses of bleak reveal that (1) bleak studied on the same spawning ground still did not reach full gut content scores on average even in the evening with some having an almost empty stomach^22^ and (2) bleak had empty gut content scores before sunrise^24^, suggesting possible motivation for nocturnal foraging. Hence, predator avoidance hypothesis seems to explain the observed spawning behaviour better than the predator satiation hypothesis. Furthermore, because of an apparent mismatch between bleak and egg presence, predator swamping hypothesis cannot be considered as an explanation^12,37^.

The presence of asp females may not be a direct indicator of egg deposition. The egg distribution in time is based on the model, which may have caused slightly different egg distribution compared to the real distribution in the studied system. Although observational data suggest that there is higher nocturnal spawning activity compared with daytime (Supplementary material 1), the possibility that some asp individuals use night just for gathering on the reproductive ground and mate at the end of the stay cannot be excluded. The eggs could be released in the early morning by some females, which would make foraging on their eggs possible for bleak. However, the majority of asp females depart before sunrise^19^ and therefore, even if spawning itself took place at the end of female visit, eggs would not be exposed to a much higher extent to bleak predation.

The reproductive behaviour of asp is not exclusively performed during a restricted time period as is described for well-studied cyprinids zebrafish *Danio rerio* or goldfish *Carassius auratus*; asp reproduction occurs not only at night, but also during the day^19,27^. The predation pressure on the studied spawning ground during the day is considerable: it was estimated that during the day 20% of asp eggs were eaten within 50 cm of their recorded drifting trajectory^22^. Therefore, it seems that female asp that time their spawning during the daytime period have lower reproductive success in comparison with females spawning at night.

Many otherwise daytime active fish species spawn at night or dusk, presumably attempting to avoid egg-eating predators^12,38–40^. This observed pattern could have evolved in the past and does not necessarily represent a novel adaptation to predation threat represented by egg-eating predators in the given environment. Comparison of reproductive timing of closely related species could shed light on the origin of this phenomenon. In related cyprinids goldfish or in zebrafish in which spawning time is well described, ovulation and milt production is synchronized among fish during the night and reproduction occurs in the early morning^41–45^. However, these species spawn in macrophytes and gravel where eggs are not so easily preyed upon in comparison with asp eggs, which have a short drifting phase where they are fully exposed to bleak predation^41,43^. Spawning patterns of more closely related species than goldfish and zebrafish (for instance other species from subfamily Leuciscinae) would provide a better comparison; however, closely related species have not been studied in such detail. Common dace (*Leuciscus leuciscus*) and chub (*Squalius cephalus*) reproduction was observed during the day^21,46^, but the data collection was not designed to test day vs. night preference in spawning activity.

Evidence from asp spawning grounds with lower predation pressure on asp eggs could help to distinguish between an obligatory night spawning pattern and adaptation of the local population. However, there is a general lack of studies on the asp spawning behaviour. Data from other riverine studies suggest that asp do not have elevated activity at night during the spawning season^26^, which could mean that the population observed in this study has adapted to local high egg predation by bleak. However, the above-mentioned study is based on a few individuals only and has been conducted with a different methodology (telemetry positioning every three hours). Therefore, these data might not be precise enough to test changes during 24-hour activity. In conclusion, more evidence should be collected to directly test whether the observed night pattern is driven by predation pressure, or is the natural behaviour of asp.

The desired habitat may be partially avoided during the day and utilized at night; such behaviour was observed in many diverse taxa. Undergoing diel vertical migration in order to avoid predation has been a widely documented phenomenon in aquatic environments^47^. Low light conditions in deep aquatic habitats are chosen to avoid predators, despite costs of inhabiting lower temperatures with lower food availability and potentially a hypoxic environment^15,48,49^. In this study, asp individuals do not migrate to spawn at night for their own safety, but more likely to avoid exposure of the eggs to diurnal predators. Similar situations occur in marine environments, where fiddler crabs (*Uca pugilator*) have reproductive cycles occurring at night which facilitate safe larval dispersal from the estuary to open sea^50,51^.

Different tagging methods and data processing had to be applied for asp and bleak (a one year pause between tagging and tracking in asp vs. one day between tagging and tracking in bleak). However, we do not expect that the observed daily pattern in bleak was influenced by this methodological difference. Bleak was sufficiently large to undergo tagging; it was reported possible to PIT-tag steelhead (*Oncorhynchus mykkis*) smaller than 90 mm fork length (average bleak size 119 ± 10 mm SL)^31^. The authors believe that the recovery after tagging might have influenced the overall amount of tagged bleak that entered the tributary after surgery, but not their daily presence pattern.

Although the primary aim of this study was to address hypotheses related to reproductive timing, the egg presence model identified also other variables apart from presence of egg predators as being significant in reproductive timing. The temperature and day of the year identified in the egg presence model is well established in the literature as a driver of reproductive timing in temperate fish species^38^. Hence, this study focused on the novel findings related to mismatch between egg and predator presence in the spawning ground.

To conclude, this study provides support for the predator avoidance hypothesis explaining nocturnal spawning in many fish species. The nocturnal spawning pattern in asp can be perceived as a behavioural adaptation to the local predation threat, but further research is needed to verify this potential explanation.

## Electronic supplementary material

Fish observational data

Von-Bertalanffy growth equation
