# Supplementary material for: Nocturnal spawning as a way to avoid egg exposure to diurnal predators
Source: Sci Rep. 2018 Oct 18;8:15377. doi: 10.1038/s41598-018-33615-4 (PMC6193928; doi:10.1038/s41598-018-33615-4)
Supplement: Supplementary file 1 — Fish observational data [file 41598_2018_33615_MOESM1_ESM.pdf]

Nocturnal spawning as a way to avoid egg exposure to diurnal predators

Marek Šmejkal[a,b*], Allan T. Souza[a], Petr Blabolil[a,b], Daniel Bartoň[a], Zuzana Sajdlová[a], Lukáš Vejřík[a,b], Jan Kubečka[a]

[a] Biology Centre of the Czech Academy of Sciences, Institute of Hydrobiology, České Budějovice, Czech Republic

[b] Biology Centre of the Czech Academy of Sciences, SOWA, České Budějovice, Czech Republic

* Corresponding author: mareks1@centrum.cz

Supplementary material 1: Fish were observed during the spawning period in a defined part of the spawning ground with size 20 * 4 m. Basic characteristics of the spawning shoal size and spawning frequency were recorded by five observers. Ranges of number of fish/ spawning events per 30 minutes observation period during the day and night are presented. Initials of observers: MŠ – Marek Šmejkal, DB – Daniel Bartoň, TK – Tomáš Kolařík, ZŠ – Zuzana Šmejkalová, KM – Karlos Ribeiro de Moraes

Observer	No. of observations	Day		Night	
		No. of fish	No. of spawning events / 30 min	No. of fish	No. of spawning events / 30 min
MŠ	15	1 - 8	0 - 2	30 - 50	15 - 60
DB	10	0 - 5	0 - 1	0 - 50	0 - 30
TK	6	0 - 8	3 - 10	10 - 45	15 - 30
ZŠ	4	0 - 5	-	20 - 40	-
KM	2	10 - 15	-	20 - 25	-
