# Supplementary material for: Nocturnal spawning as a way to avoid egg exposure to diurnal predators
Source: Sci Rep. 2018 Oct 18;8:15377. doi: 10.1038/s41598-018-33615-4 (PMC6193928; doi:10.1038/s41598-018-33615-4)
Supplement: Supplementary file 2 — Von-Bertalanffy growth equation [file 41598_2018_33615_MOESM2_ESM.pdf]

Nocturnal spawning as a way to avoid egg exposure to diurnal predators

Marek Šmejkal[a,b*], Allan T. Souza[a], Petr Blabolil[a,b], Daniel Bartoň[a], Zuzana Sajdlová[a], Lukáš Vejřík[a,b], Jan Kubečka[a]

[a] Biology Centre of the Czech Academy of Sciences, Institute of Hydrobiology, České Budějovice, Czech Republic

[b] Biology Centre of the Czech Academy of Sciences, SOWA, České Budějovice, Czech Republic

* Corresponding author: mareks1@centrum.cz

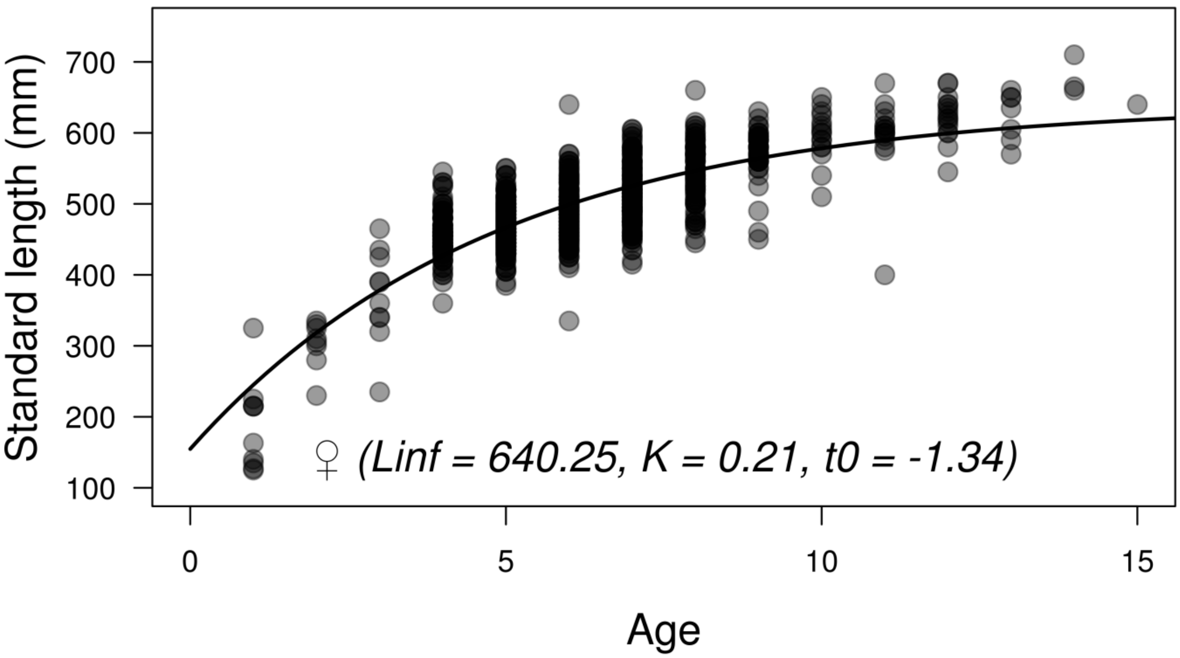

Supplementary material 2: Von-Bertalanffy growth equation used for subsequent estimation of female length in the years after tagging. In fish that have been recaptured in the year of the behavioural study, their individual predicted length was corrected based on their true length increment.
